# Upregulation of complement proteins in lung cancer cells mediates tumor progression

**DOI:** 10.3389/fonc.2022.1045690

**Published:** 2023-01-05

**Authors:** Emily K. Kleczko, Joanna M. Poczobutt, Andre C. Navarro, Jennifer Laskowski, Amber M. Johnson, Sean P. Korpela, Natalia J. Gurule, Lynn E. Heasley, Katharina Hopp, Mary C.M. Weiser-Evans, Elizabeth B. Gottlin, Ryan T. Bushey, Michael J. Campa, Edward F. Patz, Joshua M. Thurman, Raphael A. Nemenoff

**Affiliations:** ^1^ Department of Medicine, University of Colorado Anschutz Medical Campus, Aurora, CO, United States; ^2^ Department of Craniofacial Biology, University of Colorado Anschutz Medical Campus, Aurora, CO, United States; ^3^ Department of Radiology, Duke University School of Medicine, Durham, NC, United States; ^4^ Department of Pharmacology and Cancer Biology, Duke School of Medicine, Durham, NC, United States

**Keywords:** complement, NSCLC, tumor microenvironment, RNA sequencing, factor H (FH)

## Abstract

**Introduction:**

*In vivo*, cancer cells respond to signals from the tumor microenvironment resulting in changes in expression of proteins that promote tumor progression and suppress anti-tumor immunity. This study employed an orthotopic immunocompetent model of lung cancer to define pathways that are altered in cancer cells recovered from tumors compared to cells grown in culture.

**Methods:**

Studies used four murine cell lines implanted into the lungs of syngeneic mice. Cancer cells were recovered using FACS, and transcriptional changes compared to cells grown in culture were determined by RNA-seq.

**Results:**

Changes in interferon response, antigen presentation and cytokine signaling were observed in all tumors. In addition, we observed induction of the complement pathway. We previously demonstrated that activation of complement is critical for tumor progression in this model. Complement can play both a pro-tumorigenic role through production of anaphylatoxins, and an anti-tumorigenic role by promoting complement-mediated cell killing of cancer cells. While complement proteins are produced by the liver, expression of complement proteins by cancer cells has been described. Silencing cancer cell-specific C3 inhibited tumor growth *In vivo*. We hypothesized that induction of complement regulatory proteins was critical for blocking the anti-tumor effects of complement activation. Silencing complement regulatory proteins also inhibited tumor growth, with different regulatory proteins acting in a cell-specific manner.

**Discussion:**

Based on these data we propose that localized induction of complement in cancer cells is a common feature of lung tumors that promotes tumor progression, with induction of complement regulatory proteins protecting cells from complement mediated-cell killing.

## Introduction

Lung cancer is the leading cause of cancer related deaths in the US (1), with lung adenocarcinoma (LUAD) comprising approximately 40% of cases. During the past 20 years it has become apparent that cancer progression is dependent on complex interactions between cancer cells and the tumor microenvironment (TME) (2). This has led to clinical development of therapeutic agents that target the TME, such as immune checkpoint inhibitors, that have been approved for numerous malignancies. While these innovative therapies tend to provide long-lasting benefit, the response rates in unselected lung cancer patients range from 14.5% to 20% in clinical trials (3–7). Furthermore, in LUAD with mutations in tyrosine kinase receptors (e.g., EGFR) or fusion kinases (e.g., EML4-ALK), the response rate is extremely low, and these patients are treated with specific inhibitors of the driver oncogenes (8). However, the TME plays a critical role in response to these agents as well (9, 10). The fact that these LUAD therapies only work in a subset of patients despite the major therapeutic advances of the past two decades (e.g., the development of targeted therapies (11–13) and immunotherapies (14)), only highlights the need for novel therapeutic targets in order to develop combination therapies that work more effectively for LUAD patients.

The interactions between cancer cells and immune cells of the TME involves a complex cross-talk in which both cell populations undergo phenotypic changes. These effects are mediated in large part by soluble factors produced by cancer cells that recruit specific populations of innate and adaptive immune cells. These recruited cells in turn signal back to the cancer cells, activating specific receptors expressed by these cells, altering the phenotype of the cancer cells. To study these interactions, our laboratory has employed an immunocompetent orthotopic mouse model, in which murine lung cancer cells derived from C57BL/6 mice are directly implanted into the lungs of syngeneic mice (15–18). Importantly, in this model cancer cells develop in the relevant microenvironment, and interact with lung-specific stromal cells. To begin to understand how cancer cells are altered by the TME, we compared the transcriptome of cancer cells recovered from orthotopic murine tumors with identical cells grown *in vitro* using RNA-seq. We used two cancer cell lines that express oncogenic KRas, and two cell lines that have EML4/ALK as the oncogenic driver. Our data identify common responses in all the cancer cells, as well as specific changes associated with distinct oncogenic drivers. The analysis reveals that pathways regulating anti-tumor immunity are induced and include antigen presentation, interferon signaling, and the complement pathway. We have further examined how specific complement regulatory proteins that are induced *in vivo* contribute to tumor progression.

## Materials and methods

### Mice

Green fluorescent protein (GFP) – expressing mice of C57BL/6 strain (C57BL/6-Tg(UBC-GFP)30Scha/J; #0043530) and wild-type (WT; C57BL/6J; #000664) were obtained from Jackson Laboratory (Bar Harbor, ME). Experiments were performed in 8-12 week old males. Animals were bred, housed, and maintained at the University of Colorado Anschutz Medical Campus vivarium. All procedures and manipulations were performed under an approved protocol by the Institutional Animal Care and Use Committee at the University of Colorado Anschutz Medical Campus.

### Tissue culture

Cells used in this project were CMT167 cells (19) (*Kras^G12V^*; p53 WT; originally provided by Dr. Alvin Malkinson at the University of Colorado), Lewis Lung Carcinoma cells (LLC; *Kras^G12C^*; p53 mutant; purchased from ATCC), EA1 cells (*Eml4-Alk*; p53 WT, developed by Dr. Stephen Malkoski at the University of Colorado (20).; and EA2 cells (*Eml4-Alk*; p53 null; obtained from Andrea Ventura at Memorial Sloan Kettering Cancer Center (21). Cell lines were cultured in either Dulbecco’s Modified Enriched Media (DMEM; CMT167 and LLC cells; Corning) or RPMI-1640 (RPMI; EA1 and EA2 cells; Corning) supplemented with 10% fetal bovine serum (FBS; Corning) and 1% penicillin/streptomycin (Corning). Cells transfected with shRNA (see below shRNA methods sections) were cultured in media that was additionally supplemented with puromycin (2µg/mL; Sigma). Cells were cultured at 37°C in a humidified incubator with 5% CO_2_. Cells were typically cultured for a maximum of 6 weeks before thawing a new vial.

### shRNA

Lentiviral transfection of shRNAs were performed in order to knockdown expression of Factor H [TRCN0000067663 (shCfh-63); TRCN0000067664 (shCfh-64), TRCN0000067666 (shCfh-66)], CD55 [TRCN00000067573 (shCd55-73)], and the non-targeting control (NTC) empty vector pLK0.1-puro plasmid (SHC001V). All TRC1 set shRNAs and controls are expressed on a lentiviral pLKO.1-puro backbone and were obtained from the Functional Genomics Shared Resource at the University of Colorado Anschutz Medical Campus. For C3 knockdown, a GIPZ lentiviral shRNA [V2LMM_67054 (shC3-A)] and the non-targeting control (NTC) shRNA (RHS4349) were purchased from GE Dharmacon. 293T cells were used to package lentiviruses encoding the shRNAs during co-transfection with PI, PII, and PIII plasmids; Turbofect Transfection Reagent (#R0533; ThermoFisher Scientific) was used to transfect cells according to manufacturer’s protocol. Target cells were infected 48 hours later with the lentiviruses in conditioned media that was filtered through a 0.45µm filter before being added to target cells overnight. Target cells were re-incubated with viral media the following day. To increase infection efficiency, cells were incubated with polybrene (8µg/mL; Sigma) for 1 hour prior to infection and polybrene (8µg/mL) was added to the viral conditioned media. Puromycin (2µg/mL) selection began 2 days after infection. After approximately 2 weeks of selection, knockdown was confirmed *via* qPCR in pools of infected cells.

### Cell proliferation

Cells were initially plated at 5000 cells per well into a 12-well tissue culture plate and treated with either 10ng/mL of IFNγ (CMT-NTC, CMT-shCd55-73, CMT-shCfh-63, EA1-NTC, and EA1-shCfh-63 cells) or TNFα (CMT-NTC and CMT-shC3-B cells). At multiple time points from days 3-8, cells were trypsinized, washed, and resuspended in an equal volume of media. Cells were counted using a Cellometer Auto T4 (Nexcelom). N=3 wells per time point per cell line.

### Quantitative real time polymerase chain reaction

RNA was isolated using the RNeasy Mini Kit (Qiagen). cDNA was made from 1ug of RNA using qScript™ XLT cDNA SuperMix (QuantaBio) following the manufacturer’s protocol. qPCR was performed on the sample using the SYBR Green qPCR Mix (Applied Biosystems) to probe for CD55, Factor H, and C3. For the induction of C3, cells were treated for 6, 24, 48, and 72 hours with PBS (control), IL-1β (10ng/mL), IFNγ (10ng/mL), IL-22 (100pg/mL), TNFα (10ng/mL), or IL-6 (100pg/mL) before RNA isolation. To validate factor H knockdown with shRNA, cells were treated for 48 hours with 10ng/mL of IFNγ before RNA isolation. To validate C3 knockdown with shRNA, cells were treated for 48 hours with TNFα (10ng/mL) before RNA isolation. Expression was normalized to β-actin. Factor H Primers: forward 5’-GAGCCTGAGACCCAACTTCC-3’, reverse 5’-CTGTGCAACGAAGGTAGTCC-3’; CD55 Primers: forward 5’- ACCCCGGTGCATAGAGAAATC-3’, reverse 5’-GGATGACGTACTGTTGTCTTGG-3’; C3 Primers: forward 5’-GCACTTGCCTCTTTAGGAAGTC-3’, reverse 5’-CCAGCTCCCCATTAGCTCTG-3’; β-actin primers: 5’-GGCTGTATTCCCCTCCATCG-3’, reverse 5’-CCAGTTGGTAACAATGCCATG-3’.

### Orthotopic mouse model

Tumors were generated by implanting murine lung cancer cells into the lungs of syngeneic mice WT C57BL/6 or GFP-transgenic mice as previously described (22, 23). Briefly, cells were suspended in Hank’s Balanced Salt Solution (HBSS; Corning) containing 1.35 mg/mL Matrigel Basement Membrane Matrix (Corning; #354234) and injected directly into left lung. Mice were anesthetized, a skin incision was made along the left lateral axillary line, subcutaneous fat was removed to visualize the left lung, and the cell suspension was injected directly into the left lung parenchyma using a 30G needle. The incision was closed using veterinary skin adhesive or staples. For the RNA-seq experiment, cells were injected at the concentration of 1x10^5^ (LLC), 2x10^5^ (CMT167), 2x10^5^ (EA1), or 5x10^5^ (EA2) cells per mouse into GFP=transgenic mice. Mice were harvested 3 (LLC) or 4 (CMT167, EA1, EA2) weeks after injections, a single cell suspension was made of the tumor-bearing left lung, and the cells were submitted to FACS analysis. For all other experiments, cells were injected at a concentration of 5x10^5^ cells/mouse (EA1, EA1-NTC, EA1-shCfh-63) or 2.5x10^5^ cells/mouse (CMT-NTC, CMT-shCfh-63, CMT-20-039, CMT-20-041) into WT C57Bl/6 mice. Mice were harvested 2-5 weeks post implantation and tumor size was measured *via* digital calipers. For studies where mice were treated, treatments began one-week post implantation of tumor cells. The Factor H autoantibody GT103 was generated at Duke University Medical Center and was injected intraperitoneal (IP) in mice 2 times per week at 200µg/mouse (24, 25).

### Single cell isolation of cancer cells

At harvest 2-4 weeks post-implantation the lung circulation was perfused with PBS/heparin (20 U/mL; Sigma). The tumor-bearing left lungs were mechanically dissociated using a razor blade and incubated for 30 minutes at 37°C with 3.2mg/mL Collagenase Type 2 (Worthington; #43C14117B), 0.75 mg/mL Elastase (Worthington; #33S14652), 0.2 mg/mL Soybean Trypsin Inhibitor (Worthington; #S9B11099N), and DNAse I (40 µg/mL; Sigma). The resulting single cell suspensions were filtered through 70µm strainers (BD Biosciences), washed with FA3 staining buffer [phosphate-buffered saline (PBS) containing 1% FBS, 2mM EDTA, 10mM HEPES]. Samples underwent a red blood cell lysis step where for 3 minutes at room temperature the samples incubated in RBC Lysis buffer (0.15 mM NH_4_Cl, 10 mM KHCO_3_, 0.1 mM Na_2_EDTA, pH 7.2), were washed, and filtered through a 40µm strainer (BD Biosciences). Single cell suspensions were submitted to FACS or traditional flow cytometry. For RNA-seq experiments, 3-5 mice were pooled for each single cell suspension. For traditional flow cytometry experiments, each single cell suspension represents one mouse.

### FACS

Cell sorting was performed at the University of Colorado Cancer Center Flow Cytometry Shared Resource using an XDP-100 cell sorter (Beckman Coulter). The sorting strategy excluded debris and cell doublets by light scatter and dead cells by DAPI (1µg/mL). Lung cancer cells were separated from the host’s GFP-expressing cells by sorting for GFP-negative cells. Immediately after sorting cells were pelleted and frozen in liquid nitrogen in preparation for RNA extraction. The number of recovered cells ranged from 2.4x10^5^ to 15x10^5^.

### Flow cytometry

A single cell suspension was performed on EA1-NTC or EA1-shCfh-63 tumors. At sacrifice, the lungs were perfused with 5 mL of PBS/heparin (20U/mL; Sigma). The single cell suspension samples were blocked in anti-mouse CD16/CD32 (clone 93; eBioscience) at 1:200 on a rocker for 15 min at 4°C. Next, fix viability dye (LIVE/DEAD Fixable Aqua Dead Cell Stain Kit; 1:200; Invitrogen; #L34966) and conjugated antibodies were added (see Antibody Panel section below) to the single cell suspension. Cells were incubated in the dark at 4°C for 60 minutes. Cells were then resuspended in FA3 buffer and ran on the Gallios Flow Cytometer (Beckman Coulter). For compensation, single-stained beads (VersaComp Antibody Capture Bead Kit; Beckman Coulter) and a single-stained cell-mix of all samples analyzed were used. Flow cytometry was analyzed using Kaluza Analysis Software (v2.0, Beckman Coulter). Compensation was first performed on the single-stained bead controls and then confirmed using the single-stained cell mixture.

*T cell Antibody Panel: *CD8-FITC (clone 53-6.7; 1:100; Invitrogen), MHCII-Dazzle (clone M5/111.15.2; 1:250; BioLegend), CD3-AF700 (clone 17A2; 1:100; BioLegend), CD4-APC/Cy7 (clone GK1.5; 1:200; BioLegend), CD45-eFlour450 (clone 30-F11; 1:100; Invitrogen), CD4-V500 (clone RM4-5; 1:200; BD Biosciences; used only for compensation).

### Innate immune cell antibody panel

CD11b-FITC (clone M1/70; 1:100; BioLegend), Ly6C-PerCP/Cy5.5 (clone HK1.4; 1:100; BioLegend), Ly6G-PE/Cy7 (clone 1A8; 1:200; BioLegend), CD45-AF700 (clone 30-F11; 1:50; Invitrogen), SiglecF-AF647 (clone E50-2440; 1:100; BD Biosciences), CD11c-APC/Cy7 (clone HL3; 1:100; BD Biosciences), CD4-V500 (clone RM4-5; 1:200; BD Biosciences; used only for compensation).

T cell and neutrophil gating strategy used has been previously published (22, 26); modifications to the T cell gating strategy include the use of different fluorophores and the substitution of CD3 for TCRβ. Monocyte gating was performed by using a quadrant gate for CD11b+ and Ly6C+ off of the CD45+ population. The gating strategy is presented in **Supplemental Figure 1**.

### RNA sequencing

Total RNA was isolated from FACS-sorted cancer cells or pelleted cells from a dish using an RNeasy Plus Mini kit (QIAgen) following the manufacturer’s protocol and including the gDNA elimination step. The quality and quantity of RNA were analyzed using NanoDrop2000 (Thermo Scientific). For samples submitted to RNA-seq, quality and quantity of samples was additionally measured using a bioanalyzer (4150 TapeStation System; Agilent). RNA-seq library preparation and sequencing were conducted at the University of Colorado Cancer Center Genomics and Microarray Shared Resource. RNA libraries were constructed using Illumina TruSEQ stranded mRNA Sample Prep Kit (Cat# RS-122-2101). Total RNA was combined with RNA purification beads to bind PolyA RNA to oligodT magnetic beads. mRNA was eluted and converted to double stranded DNA. A-tailing, adapter ligation, and PCR amplification using 15 cycles was performed to complete the library construction. Libraries were quantitated *via* Qubit, analyzed on a Bioanalyzer Tape Station, and diluted to appropriate concentration to run on an Illumina HiSEQ 2500 High Throughput Flow Cell (CMT and LLC cells) or an Illumina NovaSEQ 6000 (EA1 and EA2 cells).

### Bioinformatic analysis

RNA-seq reads were obtained using Illumina Hi-seq analysis Pipeline. Read quality was checked using FastQC (http://www.bioinformatics.bbsrc.ac.uk/projects/fastqc). The median number of reads per condition was 24 million. Reads were trimmed for quality and adapter removal and cropped using Trim Galore on Galaxy, implementing Cutadapt (http://usegalaxy.org). Reads were aligned to the UCSC *Mus musculus* reference genome (build mm10), assembled into transcripts, and transcript abundance was estimated using Basespace Illumina Application “RNA-seq Alignment v 1.1.0” (www.basespace.illumina.com) implementing the following set of software: Isis (Analysis Software) v. 2.6.25.18, STAR (Aligner) STAR_2.5.0b, Isaac Variant Caller 2.3.13-31-g3c98c29-dirty, BEDTools 2.17.0, Cufflinks 2.2.1, BLAST 2.2.26+. Differential expression was analyzed using Basespace Illumina Application “Cufflinks Assembly & DE v2.1.0”, using standard FPKM normalization method. Pathway analysis was performed on the expression dataset of FPKM values using Gene Set Enrichment Analysis (GSEA; http://gsea-msigdb.org), with the Hallmark gene sets (50 gene sets) collection, which was converted to *Mus musculus* in R software using the msigdbr package. The metric for ranking genes was a log2 Ratio of Classes. The criteria used for significant pathways was an FDR q value less than.05. To select differentially expressed genes we used the following criteria: in a pairwise analysis, a differentially expressed gene was in the leading edge of the GSEA with a rank score of less than -1 in addition to having the FPKM value greater than 5. Further analysis of differentially expressed genes and graphing was performed in R software. RNA-seq data was deposited in the Gene Expression Omnibus in 2017 (LLC and CMT; accession number GSE100412) and 2022 (EA1 and EA2; accession number GSE204918). Additional heat maps were generated on Prism Software using a complement gene list published by Monteran et al. (27)

### Statistical analysis

Except for RNA-seq analysis described above, all analyses were performed using Prism 9 (Version 9.4.0; GraphPad Software). Data are presented as the mean and standard error of the mean (SEM). Comparisons between groups were performed by non-parametric Mann-Whitney test to determine statistical significance unless otherwise noted. Differences in survival were analyzed using the Mantel-Cox test. ROUT outlier was performed on data where Q was 1%. p-values are denoted by **(P ≤ 0.01) and ***(P ≤ 0.001).

## Results

### Transcriptional profiling of cancer cells from tumor-bearing mice

To define signaling pathways that are important for crosstalk between cancer cells and the tumor microenvironment, we sought to recover cancer cells from established lung tumors using our orthotopic model. Since cancer cells do not generally express unique cell surface markers, we injected murine lung cancer cells into the left lobe of C57BL/6-Tg(UBC-GFP)30Scha/J mice, which express GFP in all tissues. 3-4 weeks post-injection, single cell suspensions of the tumor bearing left lung were prepared and the GFP-negative population, representing cancer cells, was selectively recovered by FACS (“*in vivo*” samples). At the time of injection, cells were simultaneously passaged in culture for 3-4 weeks, in parallel to the *in vivo* experiments, and then frozen for RNA isolation (“*in vitro*” samples) (**Supplemental Figure 2A**). Examination of the GFP-negative cells under the microscope revealed a uniform morphology indistinguishable from the original population. We used 4 murine cell lines derived from the C57BL/6 strain: Lewis lung carcinoma (LLC; originally derived from a spontaneous cancer that harbors a *Kras^G12C^* mutation (28)), CMT167 cells (derived from a spontaneous cancer that harbors a *Kras^G12V^* mutation (29)), and two cell lines derived from tumors induced by injection of an Adeno-cre virus inducing *Eml4-Alk* translocation (21) (EA1/EA2). RNA-seq was performed on multiple (3–5) isolations of each sample in order to compare changes in transcriptional profiles between *in vivo* and *in vitro* samples.

By Principal Component Analysis, the *Kras* mutated CMT167 cell line clustered more closely with the *Eml4-Alk* fusion EA1 cell line, while the *Kras* mutated LLC cell line clustered more closely with the *Eml4-Alk* fusion EA2 (**Supplemental Figure 2B**). All of the *in vivo* samples for each cell line clustered closely together with the *in vitro* samples, with the exception of the LLC tumors. To further analyze the data, we performed a Spearman correlation analysis to look at the rank relationship (**Supplemental Figure 2C**). Similar to the PCA analysis, the cell lines clustered closely to each other and CMT167 cells clustered more closely to EA1 cells while LLC cells clustered more closely to EA2 cells.

### Cancer cells *in vivo* upregulate immune pathways in response to the TME

**Figure 1:**
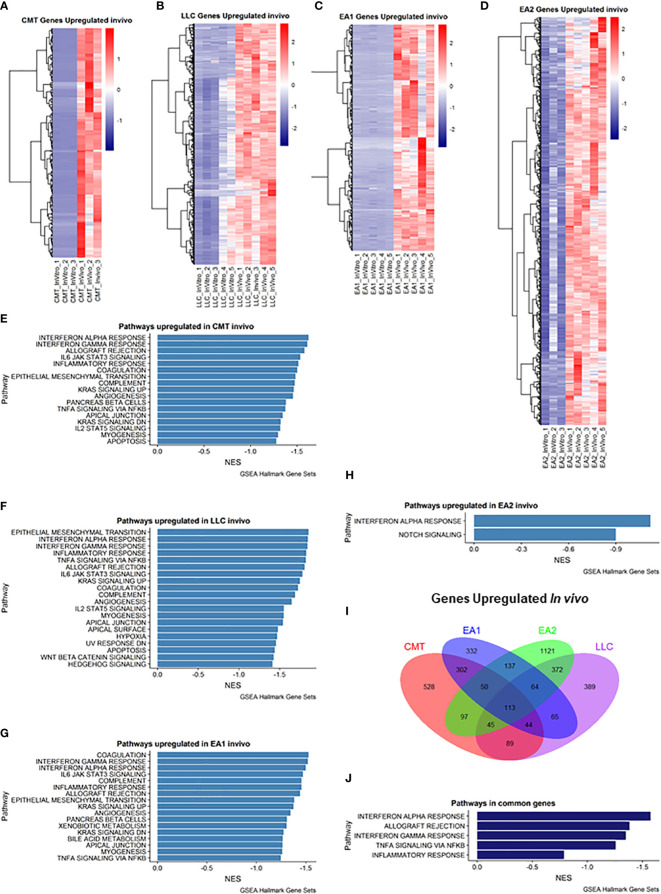
Analysis of genes upregulated *in vivo* compared to *in vitro* cells. **(A–D)**: Heat map of genes differentially upregulated in cells recovered from tumors (*in vivo*) compared to cells grown in culture (*in vitro*) for each of the cell lines: **(A)** CMT167, **(B)** LLC, **(C)** EA1, **(D)** EA2. Gene set enrichment analysis of Hallmark Gene Sets shows pathways upregulated *in vivo* compared to *in vitro* cells in CMT167 cells **(E)**, LLC cells **(F)**, EA1 cells **(G)**, and EA2 cells **(H)**. **(I)** Venn diagram shows genes commonly and/or differentially upregulated in all tumor types. **(J)** shows the common pathways upregulated in all 4 tumors. Differentially expressed genes were defined as being in the leading edge of the GSEA and had a rank score of less than -1, and the FPKM value had to be greater than 5.

We employed Gene Set Enrichment Analysis (GSEA) to examine genes that were upregulated *in vivo* in all four tumor samples compared to their *in vitro* counterparts. Differentially expressed genes were defined as being in the leading edge of the GSEA, having a rank score of less than -1, and having an FPKM value greater than 5. Each cell line had varying numbers of differentially expressed genes (**Figures 1A–D**). GSEA analysis revealed that each cell line showed induction of similar pathways including Interferon Alpha/Gamma Response and allograft rejection pathways – pathways which encompass the Kyoto Encyclopedia of Genes & Genomes (KEGG) antigen presentation and processing pathway (**Figures 1E–H**). With these criteria we identified 113 genes that were increased in all four cell lines (**Figure 1I**), and further GSEA analysis of these genes revealed 5 pathways that were upregulated *in vivo* in all 4 cell lines, including the Interferon Alpha/Gamma Response and allograft rejection pathways (**Figure 1J**). Further analysis of the differential expression reveals specific genes that are upregulated *in vivo* compared to *in vitro* cells (**Supplemental Figure 3**). Interestingly, numerous cytokines and cytokine receptors were upregulated (**Supplemental Figure 3D**), consistent with the TME altering communication between cancer cells and stromal cells.

**Figure 2:**
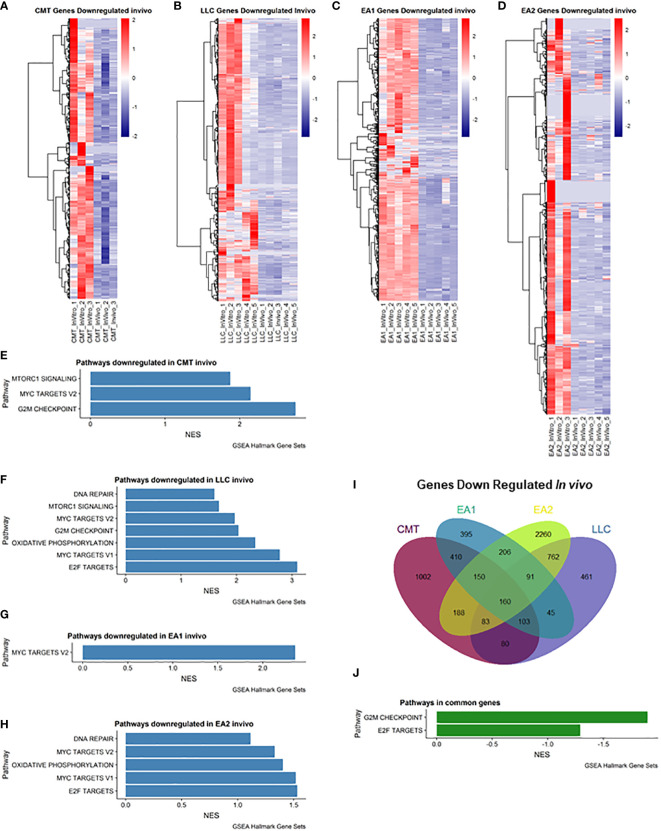
Analysis of genes downregulated *in vivo* compared to *in vitro* cells. **(A–D)**: Heatmap of genes differentially downregulated in cells recovered from tumors (*in vivo*) compared to cells grown in culture (*in vitro*) for each of the cell lines: **(A)** CMT167, **(B)** LLC, **(C)** EA1, **(D)** EA2. Gene set enrichment analysis of pathways of Hallmark Gene Sets shows pathways downregulated *in vivo* compared to *in vitro* cells in CMT167 cells **(E)**, LLC cells **(F)**, EA1 cells **(G)**, and EA2 cells **(H)**. **(I)** Venn diagram shows genes commonly and/or differentially upregulated in all tumor types. **(J)** shows the common pathways upregulated in all 4 tumors. Differentially expressed genes were defined as being in the leading edge of the GSEA and had a rank score of less than -1, and the FPKM value had to be greater than 5.

We also examined genes that were downregulated *in vivo* (**Figure 2**). Myc Targets Pathways were downregulated in all 4 cell lines *in vivo* (**Figures 2E–H**). It is interesting to note that these pathways were the only pathways downregulated in EA1 according to GSEA (**Figure 2G**). We observed 160 genes that were commonly downregulated in all 4 tumors that fell into 2 pathways – G2M checkpoint and E2F targets (**Figures 2I, J**).

### Induction of a complement signature

**Figure 3:**
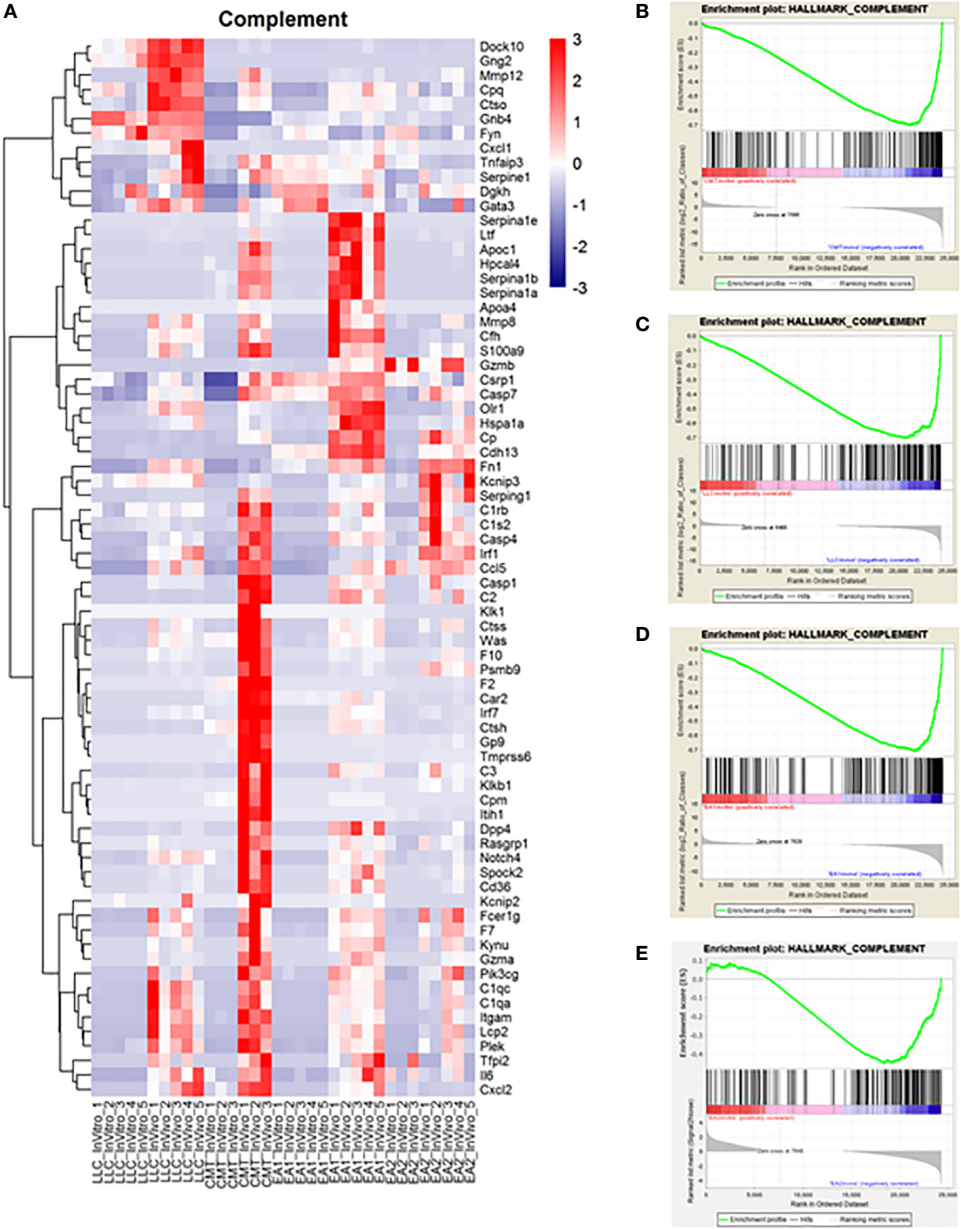
Complement proteins induced *in vivo* in murine NSCLC tumors and complement genes were found to be upregulated *in vivo* compared to *in vitro* cells. Gene Set Enrichment Analysis was performed on the data set to analyze changes in the complement pathway *in vivo* versus *in vitro*. **(A)** A heat map showing the upregulation of complement pathway genes *in vivo*. Enrichment plots for the complement pathway in CMT cells **(B)**, LLC cells **(C)**, EA1 cells **(D)**, and EA2 cells **(E)**. The “HALLMARK_COMPLEMENT” gene set from the Hallmark Gene Sets for this GSEA. The enrichment plots show the *in vitro* changes that positively correlate with complement genes on the left side of the plot in red while the *in vivo* changes negatively correlated with complement genes are on the right side in blue.

Another major pathway of interest was the complement pathway (**Figure 3**; **Supplemental Figure 4**). This pathway was more enriched *in vivo* in both CMT167 and EA1 tumors, compared to LLC and EA2, although all 4 cell lines induced multiple complement genes (17). The complement pathway is a part of the innate immune system that interacts with and regulates the adaptive immune system, and its primary role in normal physiology is to help clear pathogens (30, 31). Complement activation occurs through three major pathways: the classical pathway, the alternative pathway and the lectin pathway (32, 33). All three pathways trigger a series of proteolytic cascades that converge on C3. The cleavage of C3 results in release of C3a and the covalent fixation of C3b to the surface of target cells. Deposited C3b leads to formation of the C5 convertase that cleaves C5 to produce C5a and ultimately leads to the formation of C5b-9, designated the membrane attack complex (MAC), which can cause lysis of target cells (34, 35). Furthermore, the cleavage products C3a and C5a (anaphylatoxins) have the ability to promote tumor growth by signaling to cells in the tumor microenvironment.

**Figure 4:**
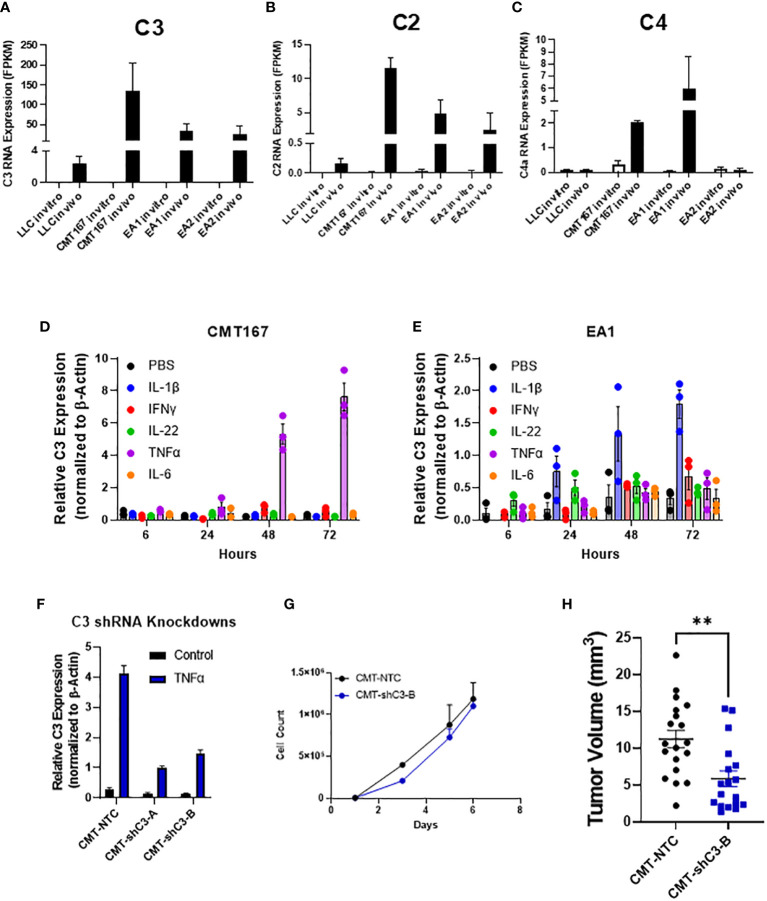
Role of C3 in tumor promotion FPKM values for C3 **(A)**, C2 **(B)**, and C4 **(C)** from our RNA-seq experiment show an induction of complement components C2 and C3 in vivo in 4 cell lines, with CMT and EA1 having an increase in C4 expression *in vivo* compared to *in vitro* samples. *In vitro*, CMT167 **(D)** and EA1 **(E)** cells were stimulated with IL1β (10 ng/mL), IFNγ (10 ng/mL), IL-22 (100 pg/mL), TNFα (10 ng/mL), IL-6 (100 pg/mL), or control (PBS) for 6, 24, 48, and 72 hours. RNA was isolated from the samples and qPCR was performed to determine what cytokines cause the induction of C3. Data were normalized to the expression of β-actin. **(F)** CMT167 cells were infected with C3 shRNA lentiviruses to knockdown down C3 expression. To confirm knockdown, cells were treated 10 ng/mL TNFα for 48 hours, RNA isolated, and qPCR performed to confirm knockdown in unselected, pooled samples. **(G)** Equal numbers of CMT-NTC or CMT-shC3-B cells were plated and proliferation was measured in the presence of TNFα over a 6 day period by quantifying cell number **(H)** 250K CMT-NTC or CMT-shC3-B cells were implanted into the left lung of C57Bl/6 mice, and established for 2 weeks before tumors were harvested and measured via digital calipers. Graph is combined of 2 independent experiments. N=9-11 per group per experiment. 2 outliers were removed using the ROUT test where Q=1%; a nonparametric Mann-Whitney was performed; **p<0.01.

While the role of complement in cancer is context dependent, our lab has previously shown that complement can drive lung cancer progression in our model by signaling to immune cells within the tumor microenvironment. We showed that complement inhibition in genetic C3^-/-^ mice and with the use of pharmacologic agents against the C3a and C5a receptors inhibited tumor growth and metastasis (17). While complement is produced largely in the liver, recent studies have demonstrated local production by other cell types, including cancer cells (36, 37). In addition to C3, the RNA-seq showed increased expression of both C2 and C4, consistent with induction of the C3 convertase (38) (**Figures 4A–C**). We hypothesized that the induction of complement proteins in cancer cells was mediated by factors produced within the TME. We therefore screened a panel of chemokines for their ability to induce both C3 in the two cell lines showing the strongest induction (CMT and EA2). As shown in **Figures 4D, E** individual factors act in a cell-specific manner to induce C3. For example, IL-1β induced C3 expression in EA1 cells, but was ineffective in the other three cell lines, whereas TNFα was the dominant inducer in CMT167. From our RNA-seq analysis, there do not appear to be significant differences in the levels of receptors between any of these cell lines, and therefore differential regulation can be attributed to differences in downstream signaling or effects of epigenetic regulation.

To confirm a role for C3 expressed in the cancer cells, we silenced expression of C3 in CMT cells using shRNA. Since expression of C3 is low in untreated cells *in vitro*, the degree of knockdown was validated in cells that had been stimulated with TNFα, which causes the greatest degree of induction (**Figure 4D**). shC3-B resulted in a greater degree of knockdown (**Figure 4F**); thus we selected these cells for *in vivo* experiments. *In vitro* proliferation assays performed in the presence of TNFα to induce C3 expression, showed no difference between control (NTC) and CMT-schC3-B cells (**Figure 4G**). To assess the role of tumor-cell derived C3 on tumor growth, CMT-NTC and CMT-shC3-B cells were implanted into the left lungs of WT mice, and tumors were harvested at 2 weeks. As shown in **Figure 4H**, silencing of C3 resulted in statistically smaller tumors.

### Silencing complementary regulatory proteins inhibits lung cancer growth

**Figure 5:**
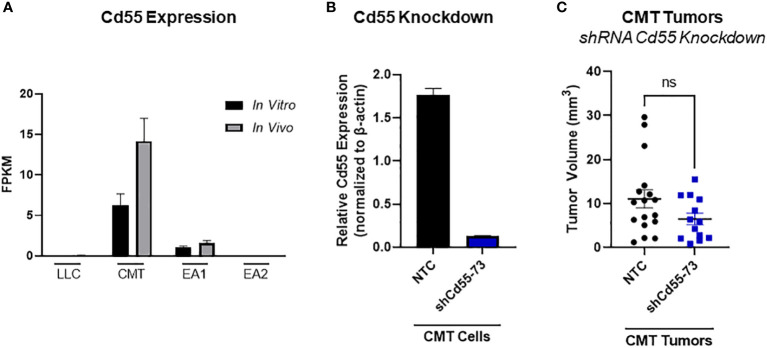
Role of CD55 on tumor progression. **(A)** Expression of complement regulatory protein CD55 from RNA-seq data comparing *in vivo* and *in vitro* samples in all 4 cell lines, where *in vivo* samples appear to induce expression compared to the *in vitro* cells. Data presented as FPKM values. **(B)** Efficiency of shRNA knockdown for CD55 in CMT cells. Pools of cells transfected with either shRNA targeting CD55 or non-targeting control (NTC) were analyzed for expression of CD55 by qRT-PCR. **(C)** 250K CMT cells silenced for CD55 or control cells were implanted into the left lung of C57Bl/6 mice. Tumor were harvested at 2 weeks post implantation and tumor volume was assessed *via* digital calipers. Graph is combined of 2 independent experiments. N=7-9 per group per experiment. 2 outliers were removed using the ROUT test where Q=1%; a nonparametric Mann-Whitney was performed; ns, not significant.

In principle, complement can play both a pro- or an antitumorigenic role (39). While production of anaphylatoxins can act to suppress anti-tumor immunity, localized complement activation resulting in the formation of the MAC can mediate cancer cell lysis and initiate an adaptive immune response. There are multiple complement regulatory proteins that act to inhibit or decrease complement activation; these proteins include Factor H (fH), CD55, and CD59 (40). We examined our RNA-seq data for expression of these proteins. *In vitro*, CD55 was only significantly expressed in CMT cells, with low to undetectable expression in all the other cell lines. CMT cells recovered from tumors (*in vivo*) showed a further induction of CD55, whereas there was no significant change in any of the other cell lines (**Figure 5A**). To determine the effects of CD55 on tumor growth, we used shRNA to knockdown expression, which resulted in a greater than 80% decrease in expression (**Figure 5B**). IFNγ has previously been reported to induce CD55 expression *in vitro* (41). Silencing CD55 had no effect on cell proliferation *in vitro* when grown in the presence of IFNγ (**Supplemental Figure 4B**). Implantation of these cells showed an inhibition of tumor growth compared to implantation of cells with a non-targeting control, though decreased tumor growth did not reach statistical significance (**Figure 5C**).

**Figure 6:**
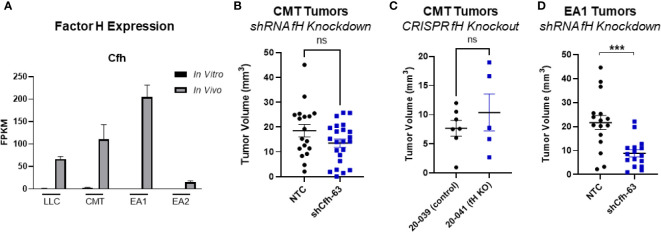
Effect of factor H on tumor progression. **(A)** Factor H (Cfh) RNA Expression *in vivo* vs *in vitro* from the RNAseq data, expressed as FPKM values. In all 4 cell lines, the *in vivo* samples appear to induce expression compared to the *in vitro* cells. **(B)** 500K CMT cells stably transfected with factor H shRNA (shCfh-63) or control cells (NTC) were implanted into the left lung of C57Bl/6 mice, established for 2 weeks, were harvested and tumors measured. Results are the combination of 3 independent experiments where n=5-10/group/experiment. **(C)** 500K CMT cells with fH deleted using CRISPR (20-041) or control cells (20-039) were implanted into the left lungs of C57Bl/6 mice, established for 2.5 weeks, and tumor volume was measured using digital calipers. N=5 or 7 per group. **(D)** 250K EA1 cells stably transfected with factor H shRNA (shCfh-63) or control cells (NTC) were implanted into the left lung of C57Bl/6 mice, established for 2.5 weeks, were harvested and tumors measured. Results are the combination of 2 independent experiments where n=6-10/group/experiment. 1 outlier was removed using the ROUT test where Q=1%. A nonparametric Mann-Whitney test was performed on all experiments; ns, not significant; ***p<0.0001.

Another complement regulatory protein that has been implicated in tumor control is factor H (42, 43). Factor H (encoded by the gene *CFH*) regulates the alternative complement pathway (AP) by serving as a decay accelerator of the AP convertases and a cofactor for factor I mediated cleavage of C3b, leading to the inhibition of both C3 and C5 convertases and downregulation of complement activation. fH was expressed at very low levels in all 4 cell lines *in vitro*, but was induced *in vivo*, with EA1 cells showing the greatest induction (**Figure 6A**). We screened the same panel of factors for their ability to induce CFH. IFNγ led to an induction of factor H RNA expression in both CMT167 and EA1 cells at 48 hours (**Supplemental Figures 5E**, **F**).

To define the role of these proteins on tumor growth, we knocked down fH in CMT cells using shRNA. (**Supplemental Figure 4A**), This had no effect on proliferation *in vitro* in the presence of IFNγ (**Supplemental Figure 4C**) and we observed no difference in tumor volume *in vivo* between the knockdown and control cells (**Figure 6B**). To confirm that this was not a result of incomplete knockdown, we used CMT167 cells that were knocked out for fH using CRISPR (44). Similar to the knockdown experiment, we observed no difference in tumor volume between factor H knockout and control tumors (**Figure 6C**).

Since fH is also robustly induced in EA1 cells, which harbor the *Eml4-Alk* fusion, we examined the role of this protein in a second cell line using shRNA. Since fH expression is low *in vitro*, we validated the degree of knockdown by stimulating cells with IFNγ, which has been shown to induce expression (45). We selected the knockdown cells that had the greatest inhibition of fH expression (designated EA1-shCfh-63; (**Supplemental Figure 4A**). *In vitro*, the proliferation of EA1-shCfh-63 cells in the presence of IFNγ was not significantly different from non-targeting control cells (EA1-NTC; **Supplemental Figure 4D**). However, upon implanting these cells orthotopically into the left lung of wild-type (WT) C57BL/6J mice, we observed a significant decrease in tumor volume in EA1-shCfh-63 tumors compared to control tumors (**Figure 6D**).

To confirm a role of fH in this cell line, we examined the effect of a therapeutic antibody targeting cancer cell-specific fH, GT103 (24, 25). The human version of this antibody is currently under clinical trials in advanced stage lung cancer (NCT04314089). The mouse version of this antibody recognizes the a common epitope ini mouse fH (**Supplemental Figure 5A**) We injected EA1 cells into the left lungs of WT mice, and began treating mice after one week with either GT103 or a control antibody. We observed significant (p=0.0012) increased survival in mice treated with the fH antibody compared to control-treated mice (**Supplemental Figure 5B**). In an attempt to quantify localized complement activation in the tumors we used immunostaining with anti-C3b and anti-C3d antibodies. While specific staining was observed, this was patchy, and there were no consistent differences between EA1-NTC and EA1-shCfh-63, or control and GT103 treatment (**Supplemental Figures 6A, B**). Attempts to stain with antbodies against components of the MAC complex were unsuccessful (data not shown).

Finally, we examined changes in the tumor microenvironment in the setting of fH knockdown. After tumors established for 3 weeks, single cell suspensions were made from the left lung/tumors of mice with both EA1-NTC and EA1-shCfh-63 tumors and were analyzed by flow cytometry. We did not observe significant changes in total T cells (CD3^+^) or in CD4^+^ or CD8^+^ T cells (**Supplemental Figures 7A–D**). However, we did observe changes in innate immune populations, with significant increases in monocytes and a non-significant trend towards increased neutrophils observed in EA1-shCfh-63 tumors (**Supplemental Figures 7E, F**).

## Discussion

Tumor progression involves complex interactions between cancer cells and the surrounding population of inflammatory, immune, and vascular cells. Cancer cells *in vivo* receive signals through cytokines and growth factors produced by stromal cells that may influence the phenotype of the cancer cell. While there has been extensive characterization of lung cancer cells *in vitro*, there is less known regarding how the microenvironment alters the properties of these cells. In this study we have used an innovative approach to recover cancer cells from an orthotopic immunocompetent mouse model of NSCLC. Recovering GFP-negative cells from a single cell digest of a tumor allowed us to recover a pure population of cancer cells that is not dependent on maintaining expression of a specific marker *in vivo*. Furthermore, the cancer cells in the tumor are in contact with populations of stromal cells that are present in the lung, which is the relevant microenvironment. Data from the RNA-seq experiment reveals the strong influence the tumor microenvironment has on the transcriptional profile of cancer cells. While we cannot determine which cells of the TME most strongly influence tumor cells, this study offers more evidence to support the importance of the TME. We employed four different cell lines, encompassing two oncogenic drivers that are common in human LUAD (KRas mutations and EML4/ALK fusions). Many of the pathways that were upregulated *in vivo* in all of the cell types are pathways that crosstalk with the immune system (e.g., antigen presentation pathway, interferon pathways). We have previously demonstrated that responsiveness to IFNγ and induction of MHCII in the tumor cells are important determinants of response to anti-PD-1 therapy in KRas-driven lung tumors (15, 16). Interactions with the immune system also appears critical in mediating the response to tyrosine kinase inhibitors in ALK positive lung cancer (46). Similarly, common pathways that were downregulated in all four cell lines, including G2M checkpoint and E2F targets, suggest that the cancer cells grow more slowly *in vivo*, presumably due to signals originating from the TME inhibiting proliferation. Our data demonstrate differences in gene expression with different cancer cells. This will likely be a result of differences in the composition of the TME in different cancers. For example, we have observed fewer T cells and less T cell activation infiltrating LLC tumors compared to CMT167 tumors (16, 18). This is consistent with differences in the response to immune checkpoint inhibitors, with CMT167 cells being responsive and LLC cells being resistant (18).

We and others have previously demonstrated that complement is activated in the setting of cancer, and that complement inhibitors have an effect in preclinical models (17),. While complement proteins are typically produced in the liver, other cells including tumor cells can express them. Our RNA-seq analysis identified complement as a pathway that was upregulated in most of our tumor models. We propose that complement activation in cancer cells occurs in response to signals from surrounding stromal cells. This results in induction of both C3 and components of the C3 convertase (C2, C4), leading to production of C3a and C5a, which could increase recruitment of immunosuppressive populations such as tumor associated macrophages, and myeloid suppressor cells. Based on our *in vitro* experiments that stimulate cancer cells, it appears that individual factors act in a cell-specific manner; in CMT cells, TNFα is the dominant factor, whereas in EA1 cells IL-1β is the most potent. However, inducing C3 expression appears to be a critical event in cancer progression as silencing C3 inhibits tumor progression. Since complement activation can ultimately lead to MAC mediated cancer cell lysis, we propose that induction of complement regulatory proteins protects cancer cells and targeting these proteins will inhibit tumor growth.

One of the most unexpected findings of the RNA-seq data is that while some of the pathways were upregulated in all or multiple cell lines, not all the same genes within these pathways are upregulated in all cell lines. The genes within these pathways appear to be differentially upregulated in different cell lines. This suggests that cells likely have multiple ways to achieve the same outcome through redundancies in pathways. Moreover, this study shows that complement regulators play a role in lung cancer progression in our mouse model. Complement regulators may function within the tumor cell to prevent the development of MAC and prevent tumor cell lysis. Using immunostaining we were not able to detect significant increases in complement activity in the setting of inhibiting fH. There are several potential reasons for this. One possibility is that if activation of MAC and subsequent lysis of cancer cells occurs, these cancer cells would be eliminated; in fact, the cancer cells remaining would be predicted to be insensitive to complement regulation. In addition, since our staining is at a single time point, it may not capture the temporal features of complement activation. However, we cannot rule out effects of targeting fH that are independent of the complement pathway, and future studies will be required to examine this. Unfortunately, we were not able to confirm an increase of MAC due to limited reagents (e.g., antibodies that do not work well). Moving forward it will be paramount to confirm MAC development in both our fH and CD55 knockdown cells to verify the mechanism through which tumor growth is inhibited. Similarly, CD55 has been shown to be elevated in many cancers, including in human lung cancer (47), and has been previously shown to promote tumorigenesis by increasing cell survival and proliferation pathways independent of the complement pathway, by leading to activation of pathways including, but not limited to SRC, NF-κB, MAPK, JNK, and JAK/STAT (48). While we did not observe any effects of CD55 silencing on cell proliferation, additional studies are required to examine effects of CD55 on other signaling pathways.

While inhibiting fH in patients may not only cause direct cancer cell death, it may also promote an adaptive immune response, important for durable anti-tumor immunity. More research is needed to fully understand the role of complement regulatory proteins, but these data suggest that these regulatory proteins in cancer play an important role in tumor growth. Future investigation into combining the inhibition of complement regulatory proteins with other strategies will hopefully yield synergistic tumor growth inhibition and improve patient survival.

## Data availability statement

The datasets presented in this study can be found in online repositories. The names of the repository/repositories and accession number(s) can be found below: https://www.ncbi.nlm.nih.gov/geo/, GSE100412, https://www.ncbi.nlm.nih.gov/geo/, GSE204918.

## Ethics statement

The animal study was reviewed and approved by Institutional Animal Care and Use Committee at the University of Colorado Anschutz Medical Campus.

## Author contributions

Conceived and designed research: EK, JP, JT, RN. Developed methodology and novel reagents: EK, JP, MW-E, EG, RB, MC, EP, RN. Performed experiments: EK, JP, AN, JL. Analyzed data: EK, JP, AJ, SK, NG. Interpreted results of experiments: EK, LH, KH, MW-E, JT, RN. Prepared figures: EK, JP, AJ, RN. Drafted manuscript: EK, JP, RN. Edited and revised manuscript: EK, JP, JL, EG, RB, MC, EP, JT, RN. All authors contributed to the article and approved the submitted version.
