# Supplementary material for: Upregulation of complement proteins in lung cancer cells mediates tumor progression
Source: Front Oncol. 2023 Jan 5;12:1045690. doi: 10.3389/fonc.2022.1045690 (PMC9849673; doi:10.3389/fonc.2022.1045690)
Supplement: Supplementary file 1 [file DataSheet_1.pdf]

Supplemental Figures and Legends

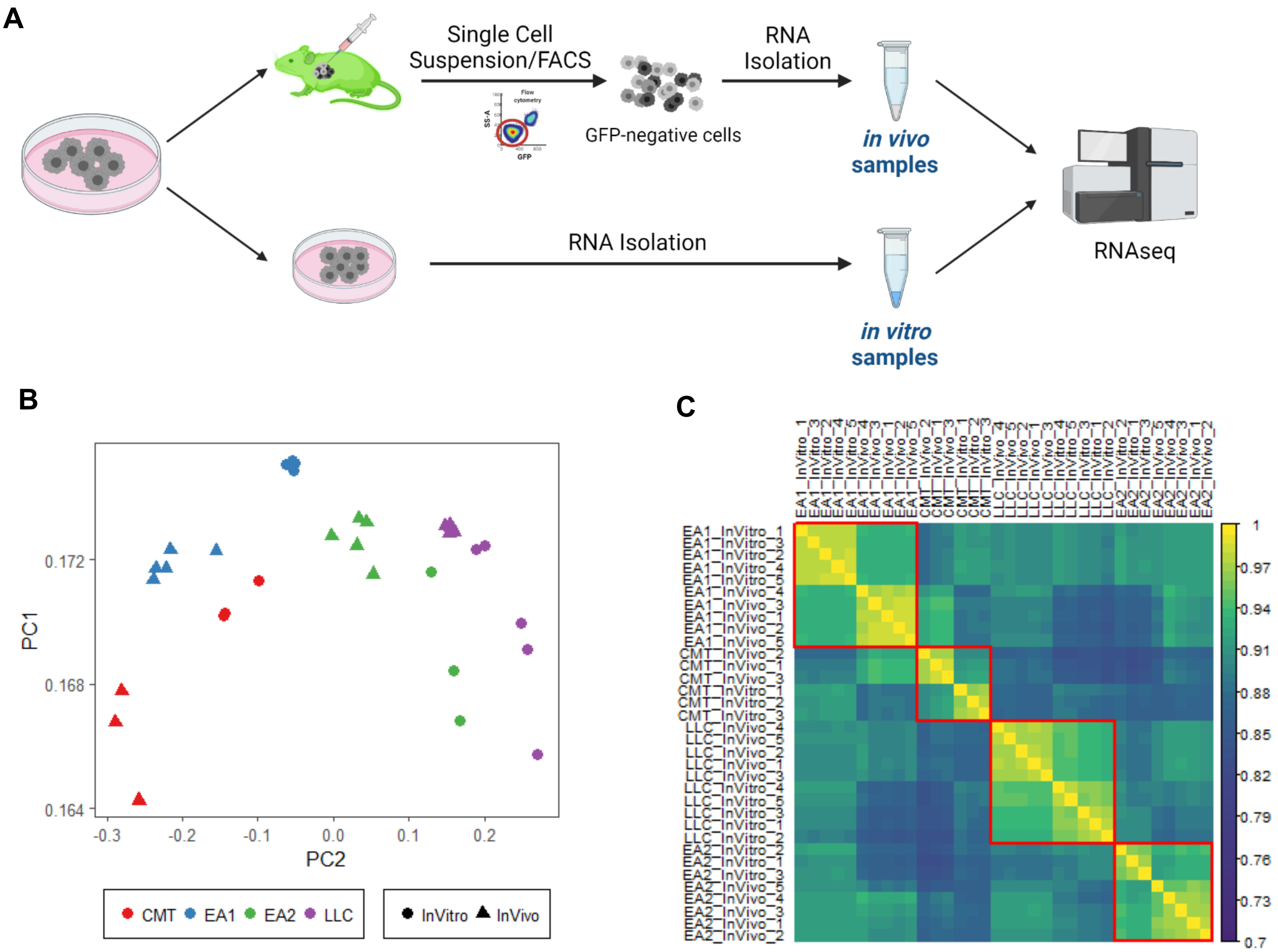

Supplemental Figure 1. Experiment Strategy for Analyzing Cancer Cells in Murine Tumors

(**A**) Experiment strategy for analyzing changes in cancer cells in murine tumors. Murine lung cancer cells (LLC, CMT, EA1 and EA2) cells were orthotopically injected into the left lung of GFP transgenic mice and tumors established for 3-4 weeks. Mice were sacrificed and single cell suspensions were made and submitted to flow cytometry cell sorting for the GFP-negative tumor cells. RNA was isolated from both the GFP-negative tumor cells and cells cultured at the time of implantation. RNA was submitted for RNA-seq analysis. Each *in vivo* sample is a pool of 3-5 mice. (**B**) Principal component analysis of the RNA-seq data for both the *in vivo* and *in vitro* samples. (**C**) Spearman correlation analysis to look at the rank relationship among *in vitro* and *in vivo* samples.

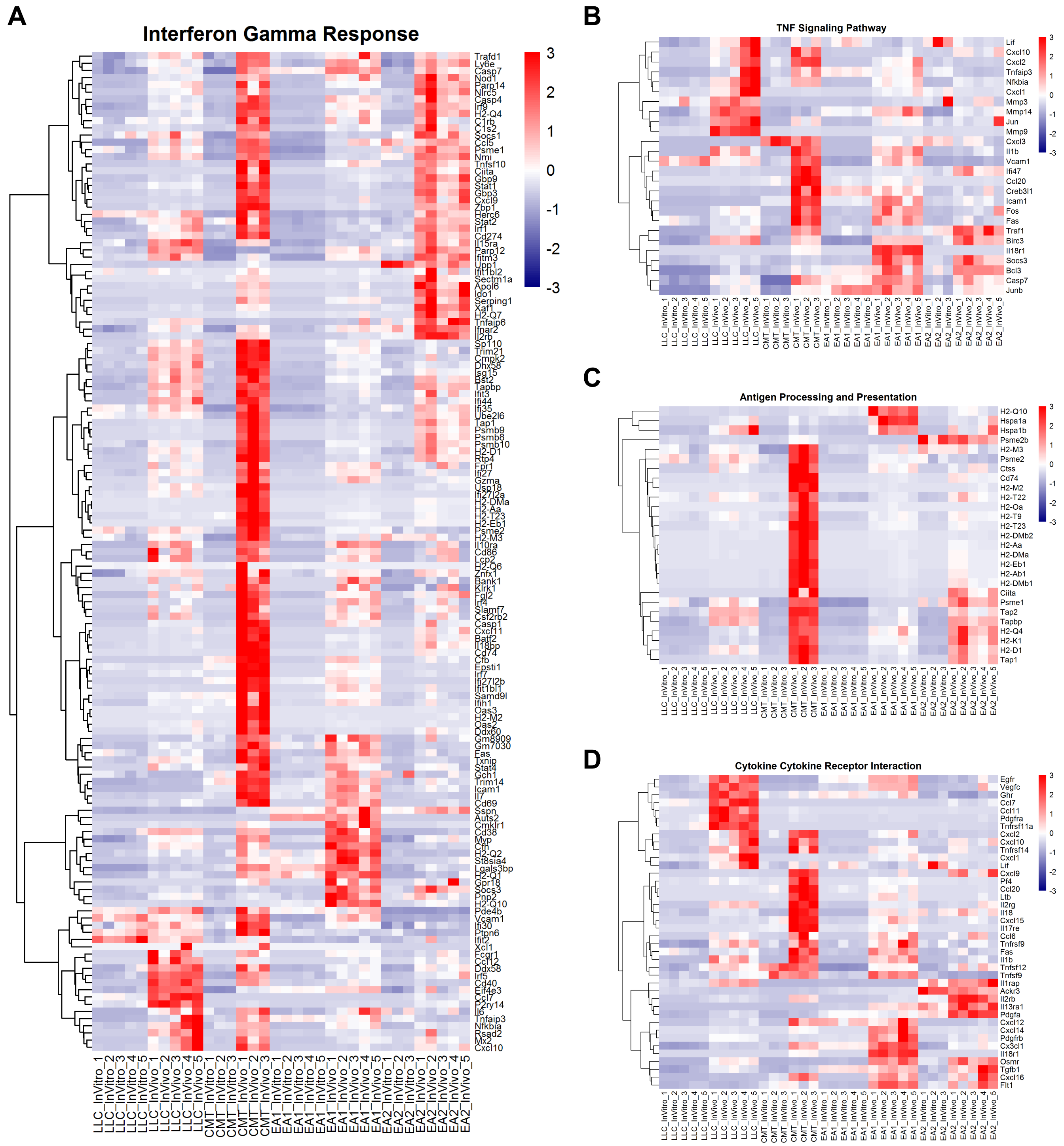

Supplemental Figure 2. Pathways induced in cancer cells in vivo

Heat maps show genes differentially expressed *in vivo* versus *in vitro* across all the tumors types. In general across all tumors, there was an induction of genes in the Interferon γ response pathway (**A**), the TNF-α response pathway (**B**), the antigen processing and presentation pathway (**C**), and the cytokine/cytokine receptor pathway (**D**). Hallmark Gene Sets were used for this gene set enrichment analysis.

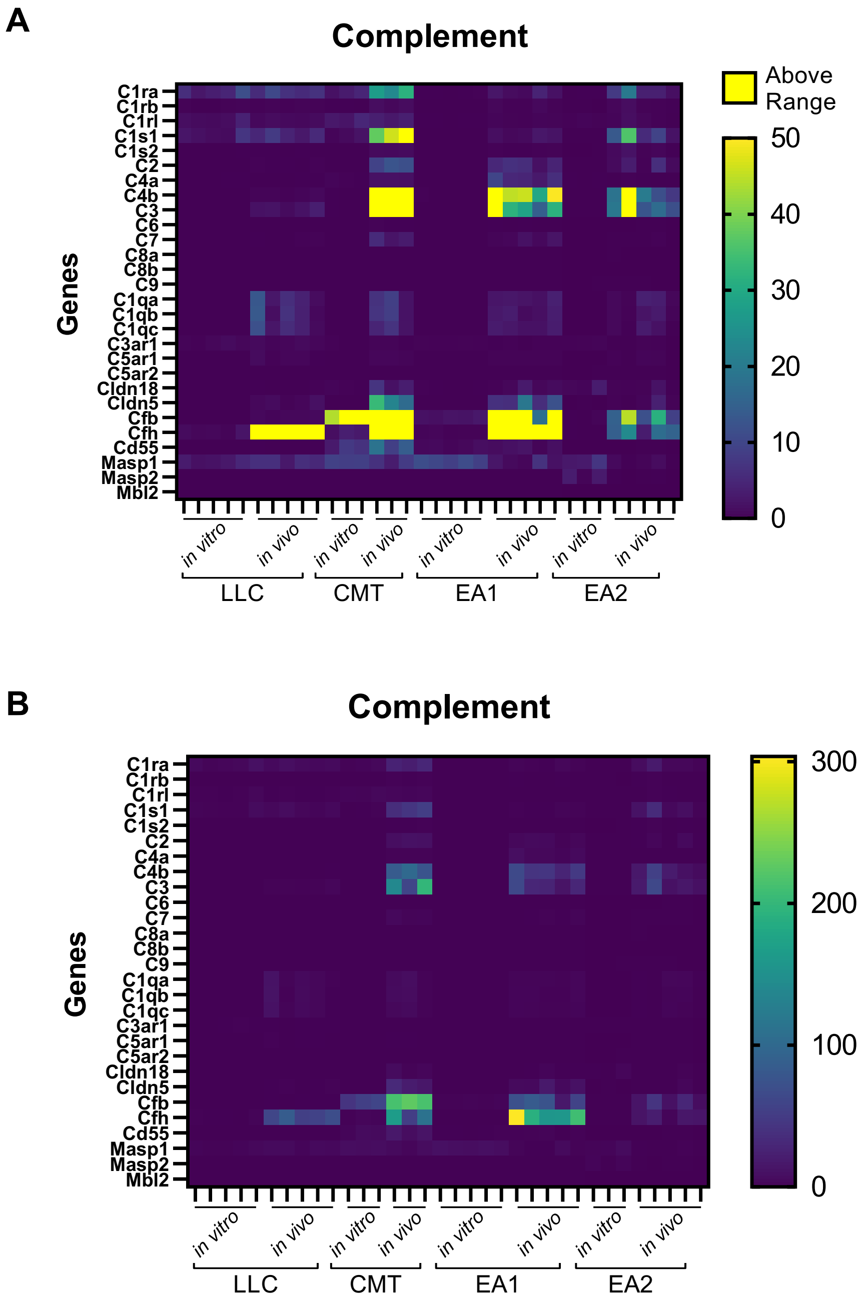

Supplemental Figure 3. Complement genes were found to be upregulated *in vivo* compared to *in vitro* cells

A heat map of complement gene expression shows the upregulation of complement pathway genes *in vivo*. **A** and **B** show the same heat map with different scales: The heat map in **A** uses a scale of 0-50, with expression levels off of the scale in yellow, while the heat map in **B** has all data points within the expression scale of 0-300. This list of complement genes were found in the publication L Monteran *et al. Nat Commun* **13**, 5797 (2022). https://doi.org/10.1038/s41467-022-33598-x. All cell lines show an increase complement gene expression *in vivo*, with CMT and EA1 cell lines having the greatest complement profile.

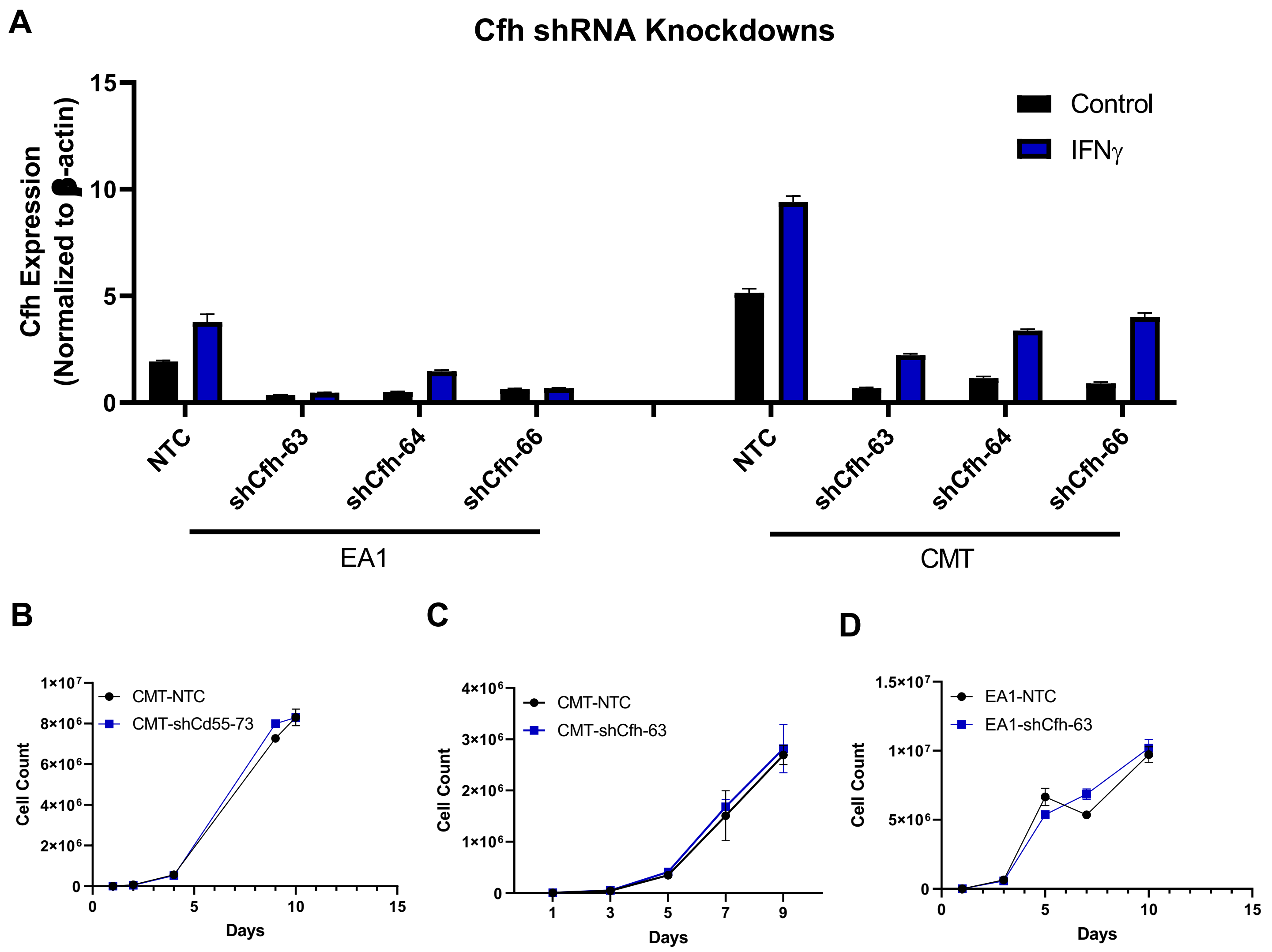

Supplemental Figure 4. Silencing fH in CMT and EA1 cells

(**A**) Quantification of factor H (fH; Cfh) expression after silencing by shRNA in EA1 cells (left) and CMT cells (right). Pooled, stable transfections of shRNA targeting Cfh or a non-targeting control (NTC) were stimulated for 48 hours with 10ng/mL IFNγ or vehicle, and factor H mRNA levels were determined by qRT-PCR. Cfh levels were normalized to β-actin. Cell proliferation assays were performed to determine growth differences between control and knockdown cells: (**B**) CMT-shCd55-73, (**C**) CMT-shCfh-63, and (**D**) EA1-shCfh-63. Cells were seeded in 12-well plates at 5000 cells/well. Cells were treated with IFNγ (10μg/ml); cells were trypsinized and counted at various days over the course of 10 days. N=3/condition. Data presented as the mean±SEM.

A

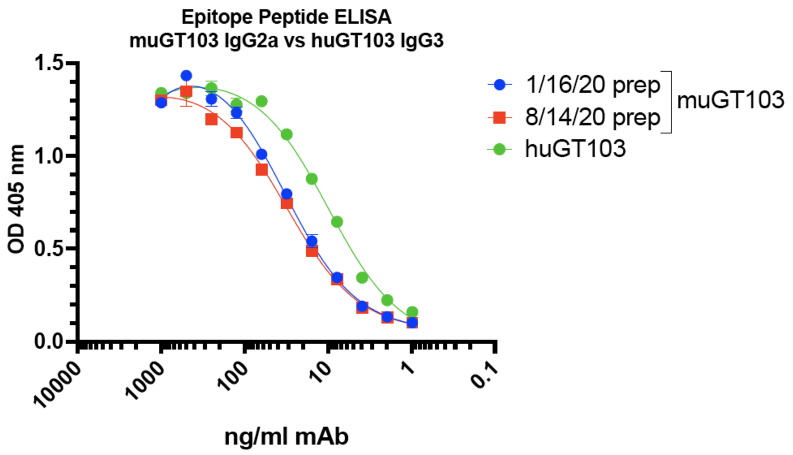


```
sp|P06909|CFAH_MOUSE      VRYECNKPLELFGQVEVMCENGIWTEKPKCRDSTGKCGPPPPIDNGDITSLSLPVYEPLS   1137
sp|P08603|CFAH_HUMAN      VRYQCRSPYEMFGDEEVMCLNGNWTEPPQCKDSTGKCGPPPPIDNGDITSFPLSVYAPAS   1132
                          ***:*..* *:**: **** ** *** *:*:*******************: * ** * *
```

B

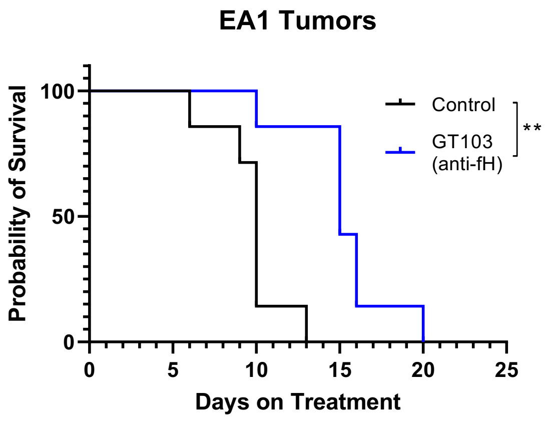

Supplemental Figure 5. Effect of Antibody Targeting fH

A. Peptide ELISA showing the human and mouse anti-CFH antibody (GT103) used in these experiments recognize the same CFH epitope, which is completely conserved in the mouse protein **B.** 5x10^5 EA1 cells were implanted into the lungs of C57Bl/6 mice. At one week post implantation, mice were treated intraperitoneally (IP) 2x/week with anti-factor H (GT103; @200µg/mouse) or control (PBS). Mouse survival was monitored over the indicated time. A Mantel-Cox test was performed to compare the survival curves; **p<0.01. N=7/group.

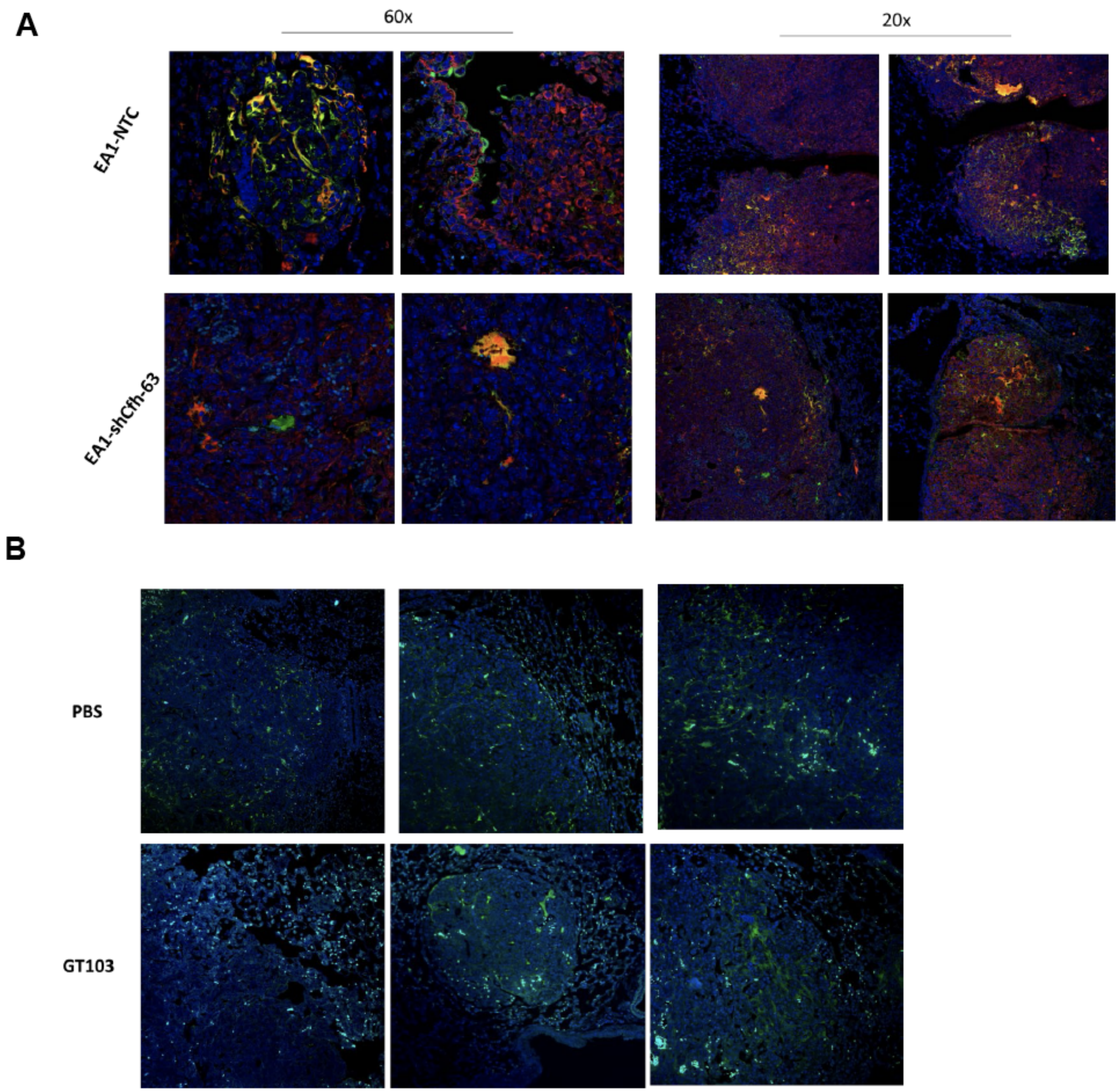

Supplemental Figure 6. Immunofluorescence for Complement Activation

Immunofluorescence staining was performed to determine changes in complement activation in EA1 factor H knockdown cells (EA1-shCfh-63; **A**) and EA1 tumors treated with the factor H antibody GT103 (**B**) compared to their respective controls. While we see positive staining, there does not appear to be any consistent staining in knockdown or treated compared to their respective controls. Blue = DAPI, Green=C3b, Red=C3d, Yellow=merged green/red. Images in **B** are at 20x.

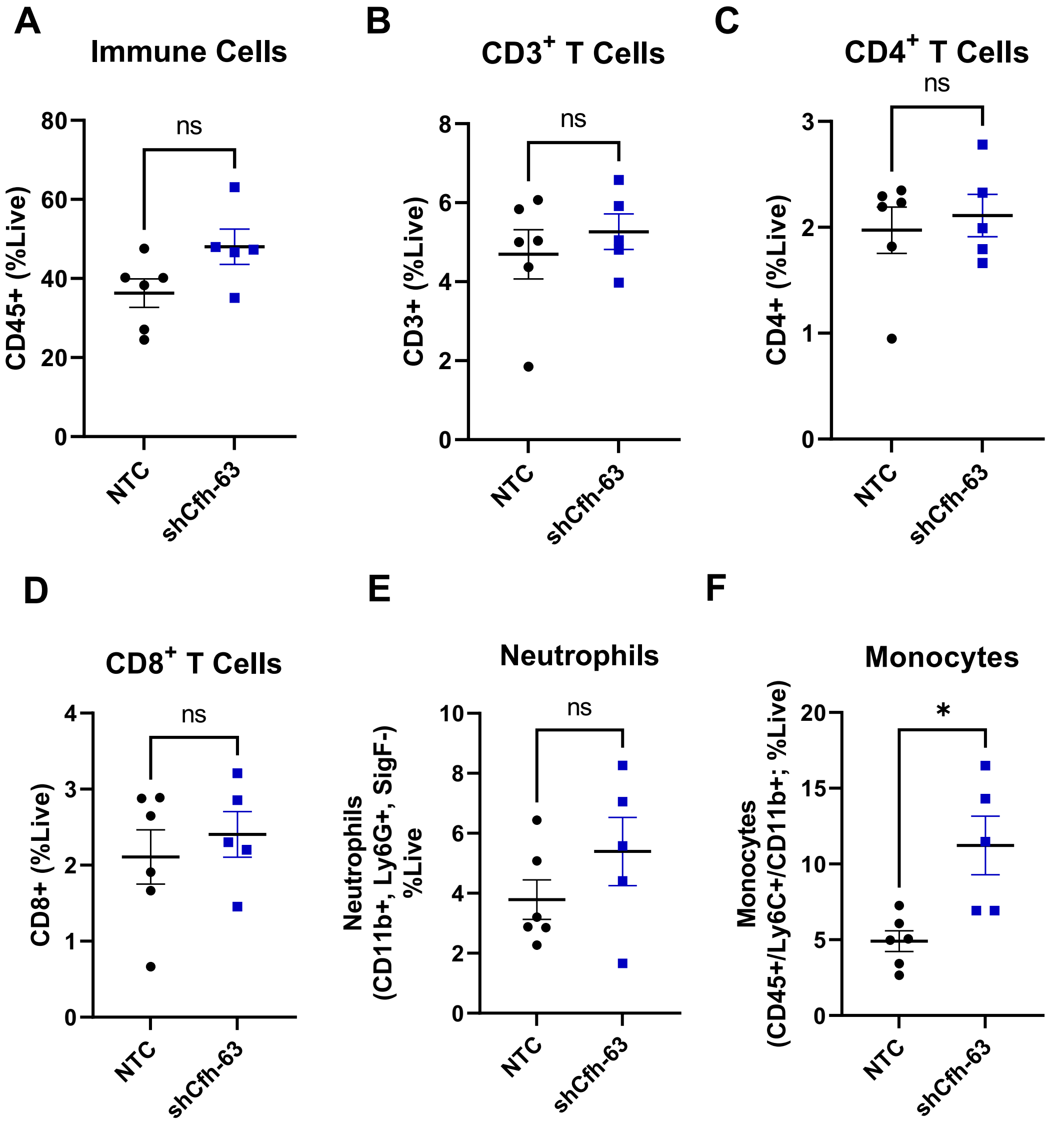

Supplemental Figure 7: Analysis of Immune cell populations in EA1 cells silenced for fH

$5x10^5$ EA1 cells fH knockdown cells (shCfh-63) or control (NTC) cells were implanted into the left lung of C57Bl/6 mice. At two weeks, single cell suspensions were made of the tumor bearing left lung lobe. Single cell suspensions were stained and analyzed on the Gallios Flow Cytometer. Populations analyzed were (**A**) Immune cells (CD45+), (**B**) CD3+ T cells, (**C**) CD4+ T Cells, (**D**) CD8+ T cells, (**E**) neutrophils (CD11b+, Ly6G+, SiglecF-), and (**F**) monocytes (Ly6C+, CD11b+). All data are shown as percent Live cells. Graphs represent 2 independent experiments; n=2-3 per group per experiment. Data are presented as the mean±SEM. A nonparametric Mann-Whitney test was performed on all experiments; ns=not significant; *p<0.05.
